# An Optimized Black-Box Adversarial Simulator Attack Based on Meta-Learning

**DOI:** 10.3390/e24101377

**Published:** 2022-09-27

**Authors:** Zhiyu Chen, Jianyu Ding, Fei Wu, Chi Zhang, Yiming Sun, Jing Sun, Shangdong Liu, Yimu Ji

**Affiliations:** 1 School of Internet of Things, Nanjing University of Posts and Telecommunication, Nanjing 210023, China; 2 School of Computer, Software and Cyberspace Security, Nanjing University of Posts and Telecommunication, Nanjing 210023, China

**Keywords:** meta-learning, adversarial attack, knowledge distillation, gradient optimization, black-box attack

## Abstract

Much research on adversarial attacks has proved that deep neural networks have certain security vulnerabilities. Among potential attacks, black-box adversarial attacks are considered the most realistic based on the the natural hidden nature of deep neural networks. Such attacks have become a critical academic emphasis in the current security field. However, current black-box attack methods still have shortcomings, resulting in incomplete utilization of query information. Our research, based on the newly proposed Simulator Attack, proves the correctness and usability of feature layer information in a simulator model obtained by meta-learning for the first time. Then, we propose an optimized Simulator Attack+ based on this discovery. Our optimization methods used in Simulator Attack+ include: (1) a feature attentional boosting module that uses the feature layer information of the simulator to enhance the attack and accelerate the generation of adversarial examples; (2) a linear self-adaptive simulator-predict interval mechanism that allows the simulator model to be fully fine-tuned in the early stage of the attack and dynamically adjusts the interval for querying the black-box model; and (3) an unsupervised clustering module to provide a warm-start for targeted attacks. Results from experiments on the CIFAR-10 and CIFAR-100 datasets clearly show that Simulator Attack+ can further reduce the number of consuming queries to improve query efficiency while maintaining the attack.

## 1. Introduction

With the recent development of deep neural networks (DNNs), people have increasingly realized that these network architectures are extremely vulnerable to attacks by adversarial perturbations [1,2,3]. By adding adversarial perturbations that humans cannot perceive to input images, DNNs [4] become unable to output correct feedback. Such a unique characteristic gives DNN robustness increasing research value. Based on how much internal network information DNNs provide, adversarial attacks are generally divided into two categories: white-box and black-box. The victim model of white-box attacks provides complete information for attackers, including the outputs of DNNs and all internal neural node gradient information [2,5]. This enables attackers to generate corresponding adversarial examples in a targeted manner. Nevertheless, such an attack background and condition do not satisfy the requirements of adversarial attacks in real environments. Thus, due to the harsh conditions of black-box attacks, with less available information, they have gradually become the recent mainstream research direction of adversarial attacks. In a black-box attack, the attacker can only obtain output information of input images from the target model, while the internal information remains hidden. Up to now, black-box adversarial attack methods proposed in academic circles have been mainly divided into three categories: query-based attacks, transfer-based attacks, and meta-learning-based attacks.

For attacks based on query, due to their high attack success rate, people have already made effort to study them under the circumstances that only the label or probability information of each input image can be obtained. Although there is still a problem in that the amount of information obtained by querying the model each time is relatively small, by using massive queries combined with a more accurate gradient estimation algorithm, attackers can still easily generate the required adversarial perturbations. In order to achieve better query results, researchers have begun to pay more attention to query efficiency problems. Various innovative methods for uncovering deeper hidden information [6,7,8,9] have emerged for increasing the query utilization rate. However, the considerable number of queries required still makes purposeful adversarial attacks detectable in real environment and signals the victim to take defensive actions.

For attacks based on model transfer, the original intention of this design was to decrease the ability of query attacks to be easily defended against. This type of black-box attack transfers part of the queries from the black-box model to the local agent model selected by the attacker in order to decrease the abnormal behavior of high-frequency queries to the black-box model [10,11,12,13,14]. Then, it uses existing white-box adversarial attack methods to generate black-box attack perturbations based on the transferred agent model. However, since the success of the attack completely depends on the similarity between the transferred local agent model and the black-box target model, the attack success rate of this method is extremely unstable. To minimize this difference between models as much as possible, a synthetic dataset training method [15,16,17] has been proposed, which cross-uses the training images and the output results of the black-box model to train the local agent model. This method also affects the black-box model’s initiation of defensive mechanisms [18,19,20]. When the training feedback of the local transferred model reaches the set threshold, the local agent model is considered a qualified imitator of the black-box target model and becomes the main target of subsequent queries. **However, this type of black-box attack is still far from reaching the attack success rate of the aforementioned query-based attack.**

For attacks based on meta-learning, the idea behind this type of attack is very novel. It optimizes and improves the shortages of query-based and transfer-based attacks. Meta-learning-based attacks use the characteristics of meta-learning and knowledge distillation to make the transferred simulator model more adaptable. This model can utilize a limited number of queries in a short time to imitate the target black-box model effectively and can quickly take on the task of acting as an accurate local agent model. As shown by Ma’s work [21], this method has the advantages of both keeping a high attack success rate and maintaining effective attack capabilities against black-box target models with defensive mechanism. However, such an attack still does not fully utilize the information of each query. For the simulator model object that is queried each time, Ma’s method [21] ignores internal information obtained by prior meta-learning and treats the model as a black-box during the entire attack process. **Therefore, fully using the internal information in the simulator model, which consumes a great amount of training cost, is worthwhile for further research.** As stated by Zhou et al. [22], the training and learning process of any model can be divided into two stages: **feature representation ability learning** and **classification ability learning**. Because the learning costs of the simulator model obtained by meta-learning are for various selected mainstream models, the feature representation ability and classification ability of these models to the training dataset have been mastered by the simulator model. When simulator attack begins, the simulator model used as a local agent model attempts to imitate the black-box target model, which has never been seen before. Referring to the transferability of a model [21], the feature representation ability of the initial simulator model is already especially similar to that of the black-box target model for the same dataset images. However, the gap between the simulator model and the black-box target model does exist in classification ability to a certain extent. Through feature extraction and visualization of the simulator model in the initial state and the model selected as the black-box target, we find that the feature attentional area of an image is almost the same between the two models. Furthermore, by output information extraction and visualization of the two models, we also observe that the initial simulator model and the black-box model have a large gap in classification ability through comparisons. Such results strongly prove the correctness and usability of the feature layer information of the simulator model.

**Figure 1:**
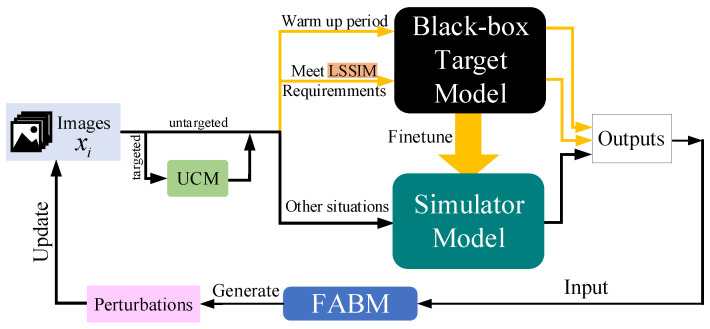
Our Simulator Attack+ iteratively updates the attack images. These images query the black-box target model for finetuning the simulator model. LSSIM gives a dynamic interval for images to visit the black-box target model for adjustment. When the simulator model becomes similar enough to the black-box model, the simulator model receives the bulk of the queries. FABM takes both local and global information into consideration to boost the attack.

**Based on this discovery, we make full use of the feature layer information of the simulator model and propose a feature attentional boosting module (FABM)**. This module strengthens the adversarial attack of perturbations, which is conducive to our attack framework to find suitable adversarial examples faster than the baseline. We add an **unsupervised clustering module (UCM)** and a **linear self-adaptive simulator-predict interval mechanism (LSSIM)** to the targeted attack to solve the cold start problem in attack situations requiring a large number of queries. Figure 1 below clearly presents the whole process of Simulator Attack+.

In this paper, for the purpose of comparing the performance of our attack framework with the baseline explicitly, we follow the settings of Ma et al. [21] to conduct adversarial attacks [8,9,23,24,25] against the same black-box target models using the CIFAR-10 [26] and CIFAR-100 [26] datasets. The experimental results show that, compared with the baseline [21], our framework achieves a certain degree of improvement in query information utilization while maintaining a high attack success rate.

The main contributions of this paper are summarized in the following points:

(1) **We find and prove that the feature representation ability of the simulator model in black-box attacks based on meta-learning is correct and usable.** The simulator model obtained through meta-learning can already represent the features and characteristics of an object in the image relatively correctly in the initial state. So the internal feature information of such a model can be used as a basis for generating and updating the perturbations that we require in the adversarial attack.

(2) **Combined with the finding of (1), we analyze and optimize Ma’s Simulator Attack [21] and propose its improved version, Simulator Attack+.** Our black-box attack framework makes specific adjustments to the three shortcomings of the baseline method by adding **FABM, LSSIM**, and **UCM** separately to solve the above mentioned problems:

- The correct feature layer information of the simulator model obtained by meta-learning is ignored in the baseline, whereas it is actually valuable for acquiring proper adversarial perturbations;
- Ma’s attack framework [21] has an imbalance in the imitation effect before and after the simulator model is fully fine-tuned;
- Adversarial perturbation changing only considers global adjustment without specialization enhancement.

(3) Conducting multi-group experiments on the CIFAR-10 and CIFAR-100 datasets, our well-designed meta-learning-based black-box attack framework greatly **improves the utilization of query information** compared with the original version, and also **raises query efficiency to a certain extent** while reducing the number of queries.

## 2. Related Works

In this section, we introduce some related work about present black-box adversarial attacks.

### 2.1. Attacks Based on Query

At present, query-based black-box adversarial attack studies follow two different directions to generate adversarial examples: score-based attacks and decision-based attacks. The former uses the probability of classification generated by the target black-box model in each query, while the latter depends on the label results of the target model outputs. Most score-based attack methods utilize specific gradient estimation calculations to find out the final adversarial perturbations. Zeroth-order optimization (ZOO) [6,27,28], inspired by derivative-free optimization (DFO) [29] and its improved versions [30], has been introduced to estimate the gradient of the target model directly for a relatively high attack success rate. However, this kind of black-box adversarial attack has to conduct a massive number of queries on the target model to collect enough information. Such information can be used to change specific pixels of an image in the attacking stage. This leads to the problem that such an attack can be easily defended against by the target model by rejecting or limiting queries in actual application scenes. In order to enhance query efficiency, strategies for focusing on the principal components of data [27], adaptive random estimation [30], involving prior gradients [9,31], active learning [32], approximating high-order gradients [33], and random search directions [34] and positions [35] have been applied. For decision-based attacks, researchers have put more focus on the use of label information [28,36] because in actual situations, classification labels are more common than score outputs. Although the information that can be obtained is sparse, query-based attacks still holds their place in black-box adversarial attack research. Several improved methods have been proposed, such as hard labeling with population-based optimization [37], an evolutionary attack algorithm [38], and a reverse boundary searching strategy [39].

### 2.2. Attacks Based on Transfer

Transfer-based attacks rely on the adversarial examples transferability theory [40]. While this type of attack can reduce the quantity of queries by consulting another designed model, the success rate is still tightly bound to the difference between the source model and the target model during the transformation period [10,12,13], which leads to the fact that such attacks may not perform well in some situations. Researchers have developed model-stealing attacks [30] and hyperparameter-stealing attacks [17] to reach higher attack success rates. For achieving a more accurate transferred model, model reconstruction [41], knockoff model [15], and local policy model [16] strategies have been published. Model reconstruction gives effective heuristic methods for reconstructing models from gradient information. A knockoff model applies queries fitting different distributions to enhance model performance. A local policy model builds the model on the basis of commonsense according to the usage of the target model and creates fake random training data for the policy model to learn decision boundaries more precisely. Nathan Inkawhich et al. [40] has pushed transfer-based attack further by using multi-layer perturbations on the basis of the extracted feature hierarchy. In this paper, we tend to train a super simulator model [21] by using the information generated from other popular recognition models to substitute for the target model. Once the simulator model has been adequately finetuned, it can perfectly imitate the black-box model, and then subsequent queries are sent to this imitator to avoid a large number of target model queries.

### 2.3. Attacks Based on Meta Learning

Model training from meta-learning has the ability to adapt to news conditions very quickly. Ma et al. [42] present MetaAdvDet with a double-network framework based on meta-learning that only requires a few queries to detect a new kind of adversarial attack. One part of this framework can learn from previous attacks, and another can do specific tasks to counter new attack methods. Du et al. [23] use meta-leaning, gradient estimation, and an auto-encoder network structure to train a meta attacker, then search for successful adversarial examples rapidly with this attacker model. Finetuning has also been inserted into this gray-box attack to improve query efficiency. Moreover, based on meta knowledge hidden in the meta-training set and meta-testing set, Ma et al. [21] then introduce a simulator model containing the features of several classic models as a substitute through meta-training and knowledge distillation. Such a simulator model structure can be defined differently by users and can also perform well when the black-box target model has some defense characteristics. Although Ma’s simulator has a rather good attack success rate for both targeted attack and untargeted attack and meets low-query demands well, the potential of simulator attacks can be pushed further by utilizing the feature layer information in a pre-trained simulator model inspired by adversarial example transferability factors study [40]. In order to strengthen adversarial attack, a random normal distribution strategy and momentum boosting strategy can be applied while generating adversarial examples against the target model. Further, for higher query efficiency, we also add an unsupervised clustering module in the simulator attacking period. In the warm-up stage, prior gradient clustering knowledge can be shared amongst all batch images, which can rapidly help part of the images finish attacking successfully. Then, we design a unique simulator-predict interval increasing mechanism to allow our simulator model to make sufficient preparation for coming queries.

## 3. Methods

To improve the query efficiency and decrease the total number of queries consumed, we propose FABM, LSSIM, and UCM and then attach these modules to our Simulator Attack+ framework. These attachments follow our discovery that similarity feature layer information between two models can help optimize the baseline method.

### 3.1. Feature Attentional Boosting Module

**Figure 2:**
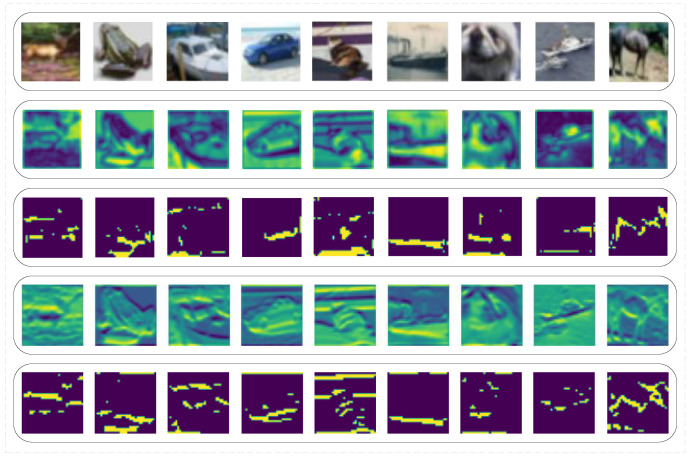
The feature layer and attentional region visualization of the first 9 images from the first batch input into the initial simulator model and the black-box model. The first line is the original images. The second line is the feature layer visualization of the initial meta simulator model. The third line is the feature attentional region of the initial meta simulator model. The fourth line is the feature layer visualization of the black-box target model (PyramidNet272). The last line is the feature attentional region of the black-box model.

**Figure 3:**
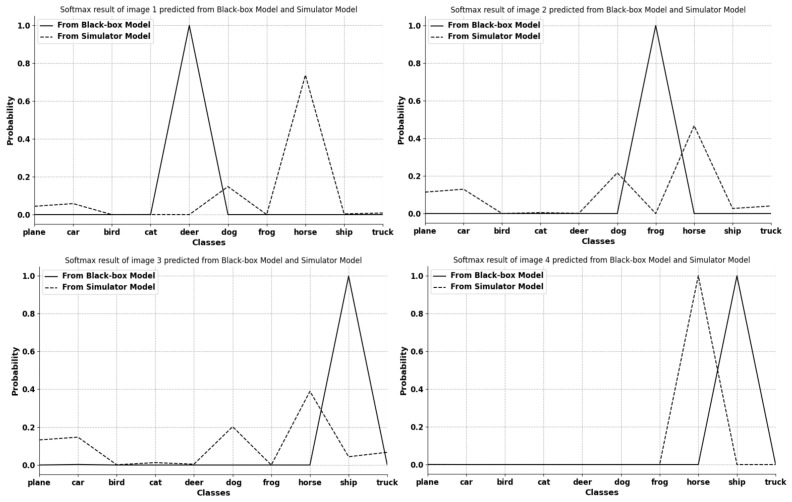
Visualization of 4 selected image softmax results from the first batch input into the initial simulator model and the black-box model. The *x* axis and *y* axis represent 10 different classes in CIFAR-10 [26] and probability values, respectively. The figure obviously shows that the classification results of the two models at the beginning are different. This indicates that the classification ability of the simulator model at this stage is not well prepared.

As a consensus, a meta simulator model trained from meta-learning can quickly imitate another model by finetuning itself and shares generality with other mainstream models. However, such a meta simulator model applied in Simulator Attack is treated the same as the black-box model. Its internal information is ignored during an attack. In order to find the usability of this information, we extract and visualize the feature layer of a simulator model (Figure 2). After comparing the feature attentional regions between the simulator model and the black-box target model, we find that the attentional areas of both models nearly overlap. Thus, we conclude that some of the feature layer information in the meta simulator model can be used in the black-box attack due to their similarity. However, Figure 3 also indicates that the classification ability varies between the initial simulator model and the black-box target model, and this classification ability is the key point that the simulator model needs to learn during the finetuning process.

In the baseline, Ma et al. [21] only use random normal distribution noise as their step-size in the image changing process. This method has strong randomness in searching for proper adversarial perturbations. Furthermore, for how to change the adversarial images properly, Ma [21] merely provides a global direction for all pixels in an image to transform without optimizing specific areas or using any corrected feature information based on prior knowledge. To improve the usage of information from queries more rationally, we add an extra adversarial effect inspired by the attention mechanism to reduce the randomness as much as possible. At the same time, such additional adversarial perturbations can create extra radical attacks on attentional regions where both the meta simulator model and the black-box model focus. We give two different options of adversarial perturbation type: normal distribution boosting and momentum boosting. Normal distribution boosting follows the method of the baseline to search for the proper adversarial perturbation randomly by using a common distribution. Because the information supplied by black-box model outputs is so sparse, we have to use additional perturbations when they are random, as in Ma et al. [21], to enhance the adversarial effect on specific regions while reducing randomness of inappropriate values in significant positions. However, different from the baseline, we consider different concrete distributions that are smoother to highlight the attentional area and keep the original distribution working as previously. As Wu et al. [40] conclude, the local oscillation of a loss surface can be suppressed by the smoothing effect. Our smoother distribution reduces harmful effects to the utmost and makes valid emphasis of attentional regions. Equations (1) and (2) show how the feature attentional boosting module works compared to a random normal distribution:

(1) $$I_{adv}=I_{adv\_prev}+P_{final},$$

(2) $$\begin{array}{c} P_{final}=\left\{\begin{array}{c} P_{org\_n},\;\;\;\;\mathrm{if}\;\mathrm{not}\;\mathrm{attentional}\;\mathrm{region};\;\\ P_{org\_n}+P_{add\_n},\;\mathrm{if}\;\mathrm{attentional}\;\mathrm{region}. \end{array}\right. \end{array}$$

where $I_{adv}$ and $I_{adv\_prev}$ refer to the attack image in the current attack step and previous attack step, respectively, $P_{final}$ represents the final adversarial perturbation in the current attack step, $P_{org\_n}$ is the original adversarial perturbation the baseline creates, and $P_{add\_n}$ is the additional adversarial perturbation belonging to another distribution we designed to strengthen the effect on the attentional region.

Momentum boosting replaces the original random normal distribution in attentional regions by an adversarial perturbation updating strategy based on momentum. This method takes both the descent direction achieved in the current attack step and all previous directions into consideration. By consulting these factors comprehensively, the additional adversarial perturbation emphasizes the adversarial effect in the attentional region and hastens the image-changing process. Equations (3) and (4) define the momentum boosting module in detail:

(3) $$\begin{array}{c} P_{final}=\left\{\begin{array}{c} P_{org\_n},\;\;\;\mathrm{if}\;\mathrm{not}\;\mathrm{attentional}\;\mathrm{region};\;\\ P_{m},\;\;\;\;\;\;\mathrm{if}\;\mathrm{attentional}\;\mathrm{region}. \end{array}\right. \end{array}$$

(4) $$P_{m}=\theta *P_{m\_cur}+\left(1-\theta \right)*P_{m\_mean},\theta \in \left(0,1\right)$$

where $P_{m}$ is the final momentum boosting adversarial perturbation added via the original random normal process, and parameter θ controls the effect generated by the current adversarial perturbation direction $P_{m\_cur}$ and the average direction $P_{m\_mean}$ calculated from all previous adversarial perturbations; the value of θ should be set in the range from zero to one.

The whole feature attentional boosting module (FABM) only works after visiting the black-box target model. As the adversarial perturbation direction obtained by the black-box target model is definitely correct, using this direction on attentional regions helps the adversarial attack succeed faster. If this module works at every stage of the attack, the total number of queries will increase instead. When the simulator model has not been fine-tuned well, it may give wrong directions for attentional module guidance. This would cause the adversarial model to require more queries to attack successfully.

### 3.2. Linear Self-Adaptive Simulator-Predict Interval Mechanism

While the simulator model acquired from meta-learning can imitate any model by finetuning itself in limited steps, the simulator model in its initial state is not well-prepared for coming queries. It still has a weak ability to give similar outputs to those of the black-box target at the early period. This leads to the fact that the first several queries to the simulator model might misdirect the changing of adversarial perturbations due to the difference between the two models, as shown in Figure 3. Thus, the whole attack process may waste queries finding the right direction, which can make the query number reasonably large. To solve this problem, we design a linear self-adaptive simulator-predict interval mechanism (LISSIM) in our simulator attack. The mechanism is divided into two parts: a linear self-adaptive function guided by a gradually increasing parameter, and a threshold value to give a maximum to the simulator-predict interval. Equation (5) describes this mechanism in detail.

(5) $$M_{int}=\left\{\begin{array}{c} \left\lfloor S_{index}/T_{adp}\right\rfloor +1,\;\\ \;\;\;\;\mathrm{if}\left\lfloor S_{\mathrm{index}}/T_{\mathrm{adp}}\right\rfloor +1\leq \mathrm{threshold};\\ M_{\max},\;\\ \;\;\;\;\mathrm{if}\left\lfloor S_{\mathrm{index}}/T_{\mathrm{adp}}\right\rfloor +1>\mathrm{threshold}; \end{array}\right.$$

where $M_{int}$ is the final interval for every visit to the black-box target model, $M_{\max}$ refers to the upper bound value of the interval for whole attack process, $S_{index}$ is the index of steps, and $T_{adp}$ is the adaptive factor we designed to control the pace of interval increases.

By using this mechanism, our simulator will have plenty of time to adjust itself to be more similar to the black-box target model. At the same time, adversarial perturbations also have enough opportunities to move further along in the appropriate direction precisely by visiting the black-box model with a high frequency during the beginning.

### 3.3. Unsupervised Clustering Module

Based on the usability of simulator model internal feature information, we add an unsupervised clustering module (UCM) as warm boot at the beginning to accelerate the whole simulator attack process. This module helps other images in the same clustering group quickly find adversarial perturbations based on the prior knowledge of clustering centers. We select a low-dimension feature clustering algorithm in this module. For the clustering mechanism, we focus on the distance between features extracted from simulator models and specific processes.

**Figure 4:**
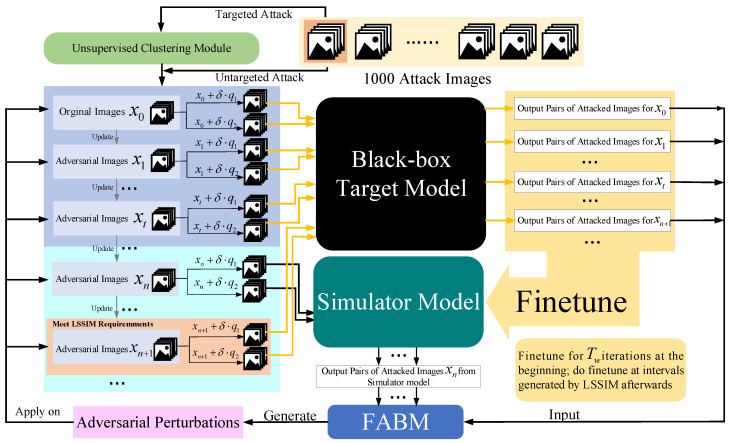
Detailed process of Simulator Attack+.

By applying an unsupervised clustering module to simulator attack, samples close to clustering centers rapidly finish their attack during the beginning of the attack process. Then we change the adversarial perturbation back to the initial state to achieve a cold boot for other unfinished images. Because these images are far from clustering centers, using prior knowledge will interference with their generating of correct adversarial perturbation. The whole process of Simulator Attack+ is exhibited in Figure 4. Firstly, the generation of adversarial perturbations relies on the estimation of image general gradient direction by adding noise $q_{1}$ and $q_{2}$.

If the attacker chooses to conduct a targeted attack, all the attack images are input into our unsupervised clustering module (UCM) to learn clustering center prior knowledge based on feature layer information. Otherwise, if the attacker chooses to conduct an untargeted attack, the attack images are immediately ready.

In the first i∈(0,t) steps, images $x_{i}$ visit the black-box target model to provide accurate information to finetune the pre-trained initial simulator model. Through this operation, our simulator model can gradually master the classification ability similar to that of the black-box target model and give precise results for any image input. When the step index meets the requirements of the interval value calculated by the linear self-adaptive simulator-predict interval mechanism (LSSIM), the attack images $x_{n+1}$ in the figure also visit the black-box target model in order to finetune the simulator model at a certain frequency. In the finetuning stage, $L_{MSE}$ influences making the simulator similar to the black-box target model. Further, other attack images such as $x_{n}$ query our finetuned simulator model for how to adjust the pixels.

The feature attentional boosting module (FABM) is utilized to enhance the adversarial effect of perturbations that are generated by the outputs of the two models. Such new perturbations are used to update the attack images from global and local perspectives in this iteration. Through constantly updating iterations, the attack images $x_{i},i\in \left(0,max\_query\right)$ finally make the black-box target model unable to recognize them correctly. Additionally, the whole simulator attack is also shown in Algorithm 1.

## 4. Experiments

### 4.1. Experiment Settings

In this section, the parameter settings of the experiment and the setup of the model are described in detail.

#### 4.1.1. Dataset and Target Models

Experiments in this paper are conducted on the CIFAR-10 [26], CIFAR-100 [26] datasets. CIFAR-10 consists of 60,000 32*32-pixel colored images from 10 classes. CIFAR-100 is composed of 100 classes of images with 600 images per class. For comparison with the work of Ma et al. [21], we also use 1000 randomly selected test images from validation sets of CIFAR-10 and CIFAR-100 for evaluating our improved attack method. For the black-box targets, we select the sames models as Ma et al. [21] and Yan et al. [43]: (1) PyramidNet+Shakedrop network with 272 layers (PyramidNet-272) [44,45] trained by AutoAugment [46]; (2) GDAS [47] generated by neural architecture search with DAG involved; (3) WRN-28 [48], which contains 28 layers and possesses a wide dimension; and (4) WRN-40 [48] with 40 layers.
**Algorithm 1** Simulator Attack+ under the $l_{p}\;norm$ condition**Input:** The input image $x\in R^{D}$, where *D* means the image dimension, the label of the image *x* with groundtruth, the pre-trained simulator model M, the forward function of the black-box target model interface *f*, and the finetuning loss function $L_{MSE}$.**Parameters:** Warm-up iteration steps *t*, the adaptive predict-interval of LSSIM $M_{adp}$, Bandits Attack parameter τ, noise exploration parameter δ, Bandits prior learning rate $\eta_{g}$, image updating rate η, the momentum factor θ of FABM, group numbers $N_{group}$ of unsupervised clustering results, the center beginning perturbations $P_{centers}$, batch_size of input images, attack type $T_{attack}$, project function $f_{proj\_p}\left(\cdot \right)$, and image update function $f_{img\_upd\_p}\left(\cdot \right)$.**Output:** Adversarial image $x_{adv}$ that meets the requirements of ϵ norm-set attack, as ${∥x_{adv}-x∥}_{p}\leq \epsilon$.1:Initialize the adversarial example $x_{adv}\leftarrow x$ and the estimated gradient g←0. Initialize the simulator model M for each image. Initialize finetune dequeue D with maximum length of *t* for coming finetuning query pairs. Initialize $N_{group}$ clustering centers randomly. Initialize empty perturbation $P_{all}$, the size of which is the same as the batch. Initialize empty perturbation $P_{centers}$, which has the same size as the clustering centers.2:**if**$T_{attack}==targeted$**then**3:    The central prior knowledge of other images in the same group is found using **UCM**.4:**for**i←1toN**do**5:      $u\leftarrow N(0,\frac{1}{D}I)$6:      $q_{1}\leftarrow \left(g+\tau u\right)/{∥g+\tau u∥}_{2}$7:      $q_{2}\leftarrow \left(g-\tau u\right)/{∥g-\tau u∥}_{2}$8:      **if** $i\leq t\;or\left(i-t\right)mod\;M_{\mathrm{adp}}=0$ **then**9:             $\hat{y_{1}}\leftarrow f\left(x_{adv}+\delta \cdot q_{1}\right)$10:           $\hat{y_{2}}\leftarrow f\left(x_{adv}+\delta \cdot q_{2}\right)$11:           Append above query pairs into dequeue D.12:           **if** i≥t **then**13:                  Finetune the simulator model M with $L_{MSE}$ using the query pairs in dequeue D.14:                  Extract feature attentional region $R_{\mathrm{atten}}$.15:    **else**16:             $\hat{y_{1}}\leftarrow M\left(x_{adv}+\delta \cdot q_{1}\right)$17:             $\hat{y_{2}}\leftarrow M\left(x_{adv}+\delta \cdot q_{2}\right)$18:    $\Delta_{dir}\leftarrow \frac{L\left(\hat{y_{1}},y\right)-L\left(\hat{y_{2}},y\right)}{\tau \cdot \delta }\cdot u$19:    Use $P_{final}$ to enhance the $\Delta_{\mathrm{dir}}$ of $R_{\mathrm{atten}}$.20:    $g\leftarrow f_{proj\_p}\left(g,\eta_{g},\Delta_{dir}\right)$21:    $x_{adv}\leftarrow f_{img\_upd\_p}\left(x_{adv},\eta ,g\right)$22:    $x_{adv}\leftarrow Clip\left(x_{adv},0,1\right)$23:**return**$x_{adv}$

#### 4.1.2. Method Setting

We follow the black-box attack process of Ma et al. [21]; we divide the whole attack into two parts: training the meta simulator and using the meta simulator to attack. In the training part, we first generate the meta-train set $D_{mtr}$ and meta-test set $D_{mte}$ on query sequence data $Q_{1},\cdots ,Q_{100}$, also known as meta tasks obtained after querying other classic models. Then, ResNet-34 is selected as the backbone of the simulator model. We train the simulator to adjust its weights by the meta-train set $D_{mtr}$ and meta-test set $D_{mte}$; each of them consists of 50 query pairs. During the attacking period, we give a 10-time fine-tuning operation as a warm up for the simulator attack. After that, the fine-tuning number reduces to a random number ranging from 3 to 5 in subsequent iterations. For an untargeted attack, the victim image may be changed randomly to a class it originally did not belong to. For a targeted attack, we give two options for attackers: random or incremental targeting. Random targeting is designed to give a random target set as $y_{adv}=rand\left(N_{class}\right)$, where $N_{class}$ is the total class number, and $y_{adv}$ is the target class. Incremental targeting sets the target class as $y_{adv}=\left(y+1\right)\;mod\;N_{class}$. For evaluating the simulator attack together with the condition of conducted query number, we introduce attack success rate and average and median values of queries. Here, the whole black-box attack process has been conducted on an NVIDIA RTX 3090 GPU. On our platform, the average time required for an untargeted attack on four selected target victim models is around 10 h, and that of a targeted attack is around 72 h.

#### 4.1.3. Pre-Trained Networks

The models we select for the meta-learning of the simulator do not contain the black-box models, so we can completely show the performance of our attack method under the hardest condition. For CIFAR-10 and CIFAR-100 datasets, we choose 14 different networks as the meta-learning materials, including AlexNet, DenseNet, PreResNet, ResNeXt, etc., and their other versions. Identical to the attack evaluation logic of Ma et al. [21], we conduct attacks against defensive black-box models. However, the simulator for defensive targets is retrained without considering ResNet networks because such targets apply ResNet-50 as their backbone. If we use the same simulator model as the normal version during the experiment, it may cause inaccuracy in the end and may be incomparable to the work of Ma et al. [21].

#### 4.1.4. Compared Methods

We follow Ma’s selection in his simulator attack and choose NES [8], Bandits [9], Meta Attack [23], RGF [24], P-RGF [25], and Simulator Attack [21] as our compared attack methods, with Simulator Attack as our baseline. We extend Ma’s criteria of these attack methods and his compared experiment results to the CIFAR-10 and CIFAR-100 datasets. We give a limit of queries of 10,000 in both untargeted and targeted attacks, and set the same ϵ values in the same experiment group: 4.6 in the $l_{2}$ norm attack, and 8/255 in the $l_{\infty }$ norm attack. In the meta-learning stage, we set the default learning rate of the inner loop update as 0.01 and of the outer ones as 0.001. In the simulator-attacking stage, the default values of the image learning rate are 0.1 and 1/255 for $l_{2}$ norm and $l_{\infty }$ norm attacks, respectively. Furthermore, the prior learning rates of Bandit attacks, also known as OCO learning rates, are 0.1 for $l_{2}$ norm attack and 1.0 for $l_{\infty }$ norm attack. For simulator predict interval, we give 5 as the standard. In the fine-tuning section, the length of the fine-tuning queue is 10, and we present 10 black-box queries as the warm-up for the whole framework. The detailed information of the default parameters for Simulator Attack+ are shown in Table 1.

### 4.2. Ablation Study

#### 4.2.1. Ablation Study for Feature Attentional Boosting Module

We first compare our two methods in FABM and decide to use the momentum boosting module in the final version of our Simulator Attack+. Then, we conduct a group of experiments for our opinion by only adding FABM and adjusting the weight values of the current direction and the average direction. The range of current direction weight value is set from 0.9 to 1. Table 2 and Table 3 show results.

#### 4.2.2. Ablation Study for Linear Self-Adaptive Simulator-Predict Interval Mechanism

Table 4 clearly shows the results of experiments with Simulator Attack with different parameters of only the LSSIM module added. This module can provide considerable positive influence under proper parameter pairs when conducting targeted attacks, as such attacks use large numbers of queries.

#### 4.2.3. Ablation Study for Unsupervised Clustering Module

We conduct a targeted attack within the $l_{2}$ norm on CIFAR-10 to test the enhancement effect of our unsupervised clustering module (UCM). Table 5 shows the results compared with the baseline [21]. As the time that this attack takes is very long, we only choose one round to show the improvement.

### 4.3. Comparisons with Existing Methods

In this section, we conduct comparison experiments with our baseline Simulator Attack and other existing black-box adversarial methods. Then, we give an analysis of the results we achieve. At last, we present our tables and figures of these experiment results.

#### 4.3.1. Comparisons with Attacks on Normal Victim Models

**Figure 5:**
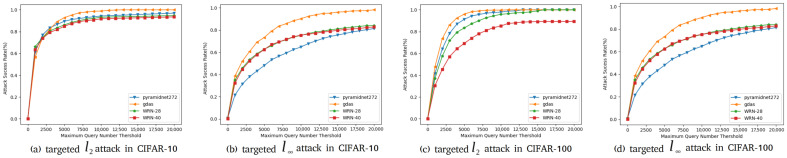
Attack success rates at different maximum query limits.

**Figure 6:**
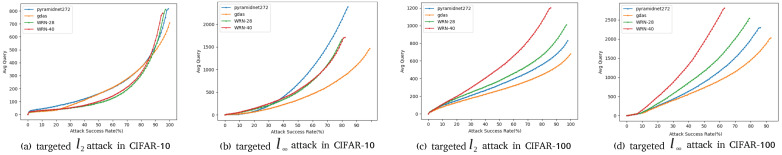
Average queries per successful image at different desired success rates.

In this part, we compare our method with our baseline Simulator Attack [21] and other classic black-box adversarial attack methods on normal victim classification models mentioned before. The models are designed without considering defensive mechanisms. Experiments are conducted on the target models we mention in Section 4.1. We show the results of these experiments on CIFAR-10 and CIFAR-100 datasets in Table 6 and Table 7. We found a decline in the success rate of attacks, mainly concentrated on targeted attacks on the CIFAR-10 and CIFAR-100 datasets. To demonstrate the effectiveness of our proposed method, Simulator Attack+, we set the maximum queries from 10,000 to 20,000, named Simulator Attack ++, and calculate the average queries when the attack success rates are close to the comparison method. To further inspect the attack success rates with different maximum numbers of queries, as shown in Figure 5 and Figure 6, we perform targeted attacks on CIFAR-10 and CIFAR-100 datasets by limiting the different maximum queries of each adversarial example.

#### 4.3.2. Comparisons with Attacks on Normal Defensive Victim Models

The results of attacks on defensive models are presented in Table 4. The defensive victim models are the same as those selected by Ma et al. [21] and include ComDefend (CD) [49], Prototype conformity loss (PCL) [50], Feature Distillation (FD) [51], and Adv Train [4]. ComDefend and Feature Distillation share a similar strategy of denoising the input images at the beginning. This operation makes sure that the images fed into the target model are their clean version. Prototype conformity loss represents a kind of loss function that is usually applied to divide the classes according to the information generated from their feature layers. For fair comparison, our PCL defensive model here is similar to Ma’s research, in that it is not trained adversarially in these experiments. Adv Train uses a min–max optimization framework to conduct adversarial training, which makes models gain strong and robust features and defensive performance. Table 8 exhibits the results of our attack against defensive models.

#### 4.3.3. Experimental Figure and Analysis

The three principal indicators of Table 6, Table 7 and Table 8, respectively, are attack success rate, average query number, and median query number. In order to compare the performance of our Simulator Attack+ with the baseline [21] and normal models under different conditions, we set $l_{2}$ and $l_{\infty }$ norm attack limits separately and maximum queries as 10,000. The results in the tables evidently show that: (1) our Simulator Attack+ method can easily obtain a reduction ranging from 5% to 10% in the average and median values of query times compared to the baseline Simulator Attack and other attacks; (2) our attack framework keeps the values of attack success rate in both types of attack closed enough to that of the original version; (3) our Simulator Attack+ also performs well when attacking black-box models with defensive mechanism.

## 5. Conclusions

In this study, we first discover the feature layer similarity of simulator models based on meta-learning. Then we propose an improved black-box attack framework, Simulator Attack+. **UCM**, **FABM**, and **LSSIM** are attached to our attack, which takes more information into consideration when searching for proper adversarial perturbations than the baseline by Ma et al. [21]. UCM in targeted attack can utilize prior gradient knowledge to accelerate the attack process. FABM can boost the perturbations in attentional regions. LSSIM helps the simulator model have a warm-start. At last, the experiment results clearly show that our Simulator Attack+ framework can use fewer queries to attack black-box target models efficiently while maintaining a relatively high attack success rate.

## Figures and Tables

**Table 1:** Default parameter settings of Simulator Attack+.

Parameters	Default	Detail
$\lambda_{1}$ for inner updating	0.1	learning rate in the inner update
$\lambda_{2}$ for outer updating	0.001	learning rate in the outer update
maximum queries	10,000	limit of queries of each sample
ϵ of $l_{2}$ norm attack	4.6	maximum distortion in $l_{2}$ norm attack
ϵ of $l_{\infty }$ norm attack	8/255	maximum distortion in $l_{\infty }$ norm attack
η of $l_{2}$ norm attack	0.1	image learning rate for updating image
η of $l_{\infty }$ norm attack	1/255	image learning rate for updating image
$\eta_{g}$ of $l_{2}$ norm attack	0.1	OCO learning rate for updating g(prior)
$\eta_{g}$ of $l_{\infty }$ norm attack	1.0	OCO learning rate for updating g(prior)
inner-update iterations	12	update iterations of learning meta-train set
simulator-predict interval	5	prediction iterations interval of simulator
warm-up iterations *t*	10	first *t* iterations of simulator attack
length of fine-tuning queue	10	maximum length of fine-tuning queue

**Table 2:** Experiments using only momentum boosting module (FABM) on CIFAR-100, untargeted attack, and $l_{\infty }$ norm.

M-Parms		Average Query				Median Query			
Cur	Avg	PN-272	GDAS	WRN-28	WRN-40	PN-272	GDAS	WRN-28	WRN-40
0.91	0.09	108	91	148	163	30	26	60	50
0.92	0.08	106	101	147	157	30	26	54	52
0.93	0.07	104	92	133	139	30	24	56	48
0.94	0.06	109	94	158	149	30	26	56	54
0.95	0.05	110	94	148	155	30	26	56	54
0.96	0.04	103	103	158	155	32	28	56	56
0.97	0.03	110	101	145	160	30	28	60	52
0.98	0.02	106	99	153	168	30	26	56	58
0.99	0.01	109	108	159	162	32	28	60	58
**Baseline**		129	124	196	209	34	28	58	54

**Table 3:** Experiments only using momentum boosting module (FABM) on CIFAR-10 against different defensive models.

M-Parms		Average Query				Median Query			
Cur	Avg	CD	PCL	FD	Adv Train	CD	PCL	FD	Adv Train
0.91	0.09	354	520	319	716	56	108	42	382
0.92	0.08	352	518	322	724	56	110	44	380
0.93	0.07	356	516	324	716	56	108	42	380
0.94	0.06	350	510	320	720	54	108	42	380
0.95	0.05	352	512	324	722	56	108	42	384
0.96	0.04	350	516	322	718	54	108	44	380
0.97	0.03	344	510	314	710	52	108	40	378
0.98	0.02	350	516	312	710	54	108	40	382
0.99	0.01	354	520	319	716	54	106	42	380
**Baseline**		388	577	350	812	60	112	44	392

**Table 4:** Experiments using only linear self-adaptive simulator-predict interval mechanism module (LSSIM) on CIFAR-100, targeted attack, and $l_{2}$ norm.

LSSIM-Parms		Average Query	Median Query
Interval Factor	Threshold	PN-272	PN-272
80	6	866	654
85	7	820	658
90	7	837	648
95	8	814	672
100	8	816	666
110	8	846	670
120	8	837	678
120	9	808	644
150	8	860	708
**Baseline**		829	644

**Table 5:** Experiments using only unsupervised clustering module (UCM) on CIFAR-10, targeted attack, and $l_{2}$ norm.

UCM	Average Query				Median Query			
Victims	PN-272	GDAS	WRN-28	WRN-40	PN-272	GDAS	WRN-28	WRN-40
SA-UCM	607	636	540	579	264	312	122	170
**Baseline**	815	715	836	793	368	400	206	245

**Table 6:** Experimental results of untargeted attacks on CIFAR-10 and CIFAR-100 datasets with a maximum of 10,000 queries.

Dataset	Norm	Attack	Attack Success Rate				Average Query				Median Query			
			PyramidNet-272	GDAS	WRN-28	WRN-40	PyramidNet-272	GDAS	WRN-28	WRN-40	PyramidNet-272	GDAS	WRN-28	WRN-40
CIFAR-10	$l_{2}$	NES [8]	99.5%	74.8%	99.9%	99.5%	200	123	159	154	150	100	100	100
		RGF [24]	100%	100%	100%	100%	216	168	153	150	204	152	102	152
		P-RGF [25]	100%	100%	100%	100%	**64**	40	76	73	62	20	64	64
		Meta Attack [23]	99.2%	99.4%	98.6%	99.6%	2359	1611	1853	1707	2211	1303	1432	1430
		Bandits [9]	100%	100%	100%	100%	151	66	107	98	110	54	80	78
		Simulator Attack [21]	100%	100%	100%	100%	92	34	48	51	52	26	34	34
		Simulator Attack+	100%	100%	100%	100%	93	**32**	**48**	**50**	**50**	**26**	**34**	**32**
	$l_{\infty }$	NES [8]	86.8%	71.4%	74.2%	77.5%	1559	628	1235	1209	600	300	400	400
		RGF [24]	99%	93.8%	98.6%	98.8%	955	646	1178	928	668	460	663	612
		P-RGF [25]	97.3%	97.9%	97.7%	98%	742	337	703	564	408	128	236	217
		Meta Attack [23]	90.6%	98.8%	92.7%	94.2%	3456	2034	2198	1987	2991	1694	1564	1433
		Bandits [9]	99.6%	100%	99.4%	99.9%	1015	391	611	542	560	166	224	228
		Simulator Attack [21]	96.5%	99.9%	98.1%	98.8%	**779**	248	466	419	469	**83**	186	186
		Simulator Attack+	95.2%	98.1%	93.0%	95.3%	781	**210**	**432**	**388**	**434**	95	**176**	**190**
CIFAR-100	$l_{2}$	NES [8]	92.4%	90.2%	98.4%	99.6%	118	94	102	105	100	50	100	100
		RGF [24]	100%	100%	100%	100%	114	110	106	106	102	101	102	102
		P-RGF [25]	100%	100%	100%	100%	54	46	54	73	62	62	62	62
		Meta Attack [23]	99.7%	99.8%	99.4%	98.4%	1022	930	1193	1252	783	781	912	913
		Bandits [9]	100%	100%	100%	100%	58	54	64	65	42	42	52	53
		Simulator Attack [21]	100%	100%	100%	100%	29	29	33	34	24	24	26	26
		Simulator Attack+	100%	100%	100%	100%	**29**	**29**	**33**	**33**	**24**	**24**	**26**	**26**
	$l_{\infty }$	NES [8]	91.3%	89.7%	92.4%	89.3%	439	271	673	596	204	153	255	255
		RGF [24]	99.7%	98.8%	98.9%	98.9%	385	420	544	619	256	255	357	357
		P-RGF [25]	99.3%	98.2%	98%	97.3%	308	220	371	480	147	116	136	181
		Meta Attack [23]	99.7%	99.8%	97.4%	97.3%	1102	1098	1294	1369	912	911	1042	1040
		Bandits [9]	100%	100%	99.8%	99.8%	266	209	262	260	68	57	107	92
		Simulator Attack [21]	100%	100%	99.9%	99.9%	**129**	**124**	196	209	34	**28**	**58**	**54**
		Simulator Attack+	100%	100%	99.8%	99.9%	133	126	**188**	**200**	**32**	32	62	60

**Table 7:** Experimental results of targeted attacks on CIFAR-10 and CIFAR-100 datasets with a maximum of up to 20,000 queries.

Dataset	Norm	Attack	Attack Success Rate				Average Query				Median Query			
			PyramidNet-272	GDAS	WRN-28	WRN-40	PyramidNet-272	GDAS	WRN-28	WRN-40	PyramidNet-272	GDAS	WRN-28	WRN-40
CIFAR-10	$l_{2}$	NES [8]	93.7%	95.4%	98.5%	97.7%	1474	1515	1043	1088	1251	999	881	881
		Meta Attack [23]	92.2%	97.2%	74.1%	74.7%	4215	3137	3996	3797	3842	2817	3586	3329
		Bandits [9]	99.7%	100%	97.3%	98.4%	852	718	1082	997	458	538	338	399
		Simulator Attack(m = 3) [21]	99.1%	100%	98.5%	95.6%	896	718	990	980	373	388	217	249
		Simulator Attack(m = 5) [21]	97.6%	99.9%	96.4%	94%	815	715	836	793	368	**400**	206	245
		Simulator Attack+	91.9%	97.7%	89.3%	89.6%	**570**	**653**	**519**	**592**	**328**	404	**166**	**194**
		Simulator Attack++	98.0%	100.0%	97.0%	94.0%	765	674	782	750	350	414	180	200
	$l_{\infty }$	NES [8]	63.8%	80.8%	89.7%	88.8%	4355	3942	3046	3051	3717	3441	2535	2592
		Meta Attack [23]	75.6%	95.5%	59%	59.8%	4960	3461	3873	3899	4736	3073	3328	3586
		Bandits [9]	84.5%	98.3%	76.9%	79.8%	2830	1755	2037	2128	2081	1162	1178	1188
		Simulator Attack (m = 3) [21]	80.9%	97.8%	83.1%	82.2%	2655	1561	1855	1806	1943	918	1010	1018
		Simulator Attack (m = 5) [21]	78.7%	96.5%	80.8%	80.3%	2474	1470	1676	1660	1910	917	957	956
		Simulator Attack+	73.8%	86.0%	71.4%	70.8%	**1231**	**1014**	**1138**	**1201**	**897**	**589**	**729**	**684**
		Simulator Attack++	79.0%	97.0%	81.0%	81.0%	2302	1307	1633	1567	1810	900	911	920
CIFAR-100	$l_{2}$	NES [8]	87.6%	77%	89.3%	87.6%	1300	1405	1383	1424	1102	1172	1061	1049
		Meta Attack [23]	86.1%	88.7%	63.4%	43.3%	4000	3672	4879	4989	3457	3201	4482	4865
		Bandits [9]	99.6%	100%	98.9%	91.5%	1442	847	1645	2436	1058	679	1150	1584
		Simulator Attack (m = 3) [21]	99.3%	100%	98.6%	92.6%	921	724	1150	1552	666	519	779	1126
		Simulator Attack (m = 5) [21]	97.8%	99.6%	95.7%	83.9%	829	**679**	1000	1211	644	**508**	706	906
		Simulator Attack+	96.2%	99.3%	92.1%	80.0%	**803**	698	**908**	**1072**	**618**	546	**630**	**780**
		Simulator Attack++	98.0%	100.0%	96.0%	84.0%	823	700	928	1130	630	550	645	852
	$l_{\infty }$	NES [8]	72.1%	66.8%	68.4%	69.9%	4673	5174	4763	4770	4376	4832	4357	4508
		Meta Attack [23]	80.4%	81.2%	57.6%	40.1%	4136	3951	4893	4967	3714	3585	4609	4737
		Bandits [9]	81.2%	92.5%	72.4%	56%	3222	2798	3353	3465	2633	2132	2766	2774
		Simulator Attack (m = 3) [21]	89.4%	94.2%	79%	64.3%	2732	2281	3078	3238	1854	1589	2185	2548
		Simulator Attack (m = 5) [21]	83.7%	91.4%	74.2%	60%	2410	2134	2619	2823	1754	1572	2080	2270
		Simulator Attack+	70.8%	78.3%	61.0%	54.6%	**1606**	**1443**	**1788**	**2011**	**1088**	**1194**	**1172**	**1322**
		Simulator Attack++	84.0%	92.0%	75.0%	60.0%	2150	1913	2305	2603	1562	1435	1765	1954

**Table 8:** Experimental results of untargeted attacks on CIFAR-10, CIFAR-100, and TinyImageNet datasets against different defensive models with a maximum of 10,000 queries. In this table, ComDefend, Feature Distillation, and Prototype Conformity Loss are referred to as CD, FD, and PCL, respectively.

Dataset	Attack	Attack Success Rate				Average Query				Median Query			
		CD [49]	PCL [50]	FD [51]	Adv Train [4]	CD [49]	PCL [50]	FD [51]	Adv Train [4]	CD [49]	PCL [50]	FD [51]	Adv Train [4]
CIFAR-10	NES [8]	60.4%	65%	54.5%	16.8%	1130	728	1474	858	400	150	450	200
	RGF [24]	48.7%	82.6%	44.4%	22.4%	2035	1107	1717	973	1071	306	768	510
	P-RGF [25]	62.8%	80.4%	65.8%	22.4%	1977	1006	1979	1158	1038	230	703	602
	Meta Attack [23]	26.8%	77.7%	38.4%	18.4%	2468	1756	2662	1894	1302	1042	1824	1561
	Bandits [9]	44.7%	84%	55.2%	34.8%	786	776	832	1941	100	126	114	759
	Simulator Attack [21]	54.9%	78.2%	60.8%	32.3%	433	641	391	1529	**46**	116	50	589
	Simulator Attack+	55.7%	76.7%	60.0%	26.6%	**388**	**577**	**350**	**812**	60	**112**	**44**	**392**
CIFAR-100	NES [8]	78.1%	87.9%	77.6%	23.1%	892	429	1071	865	300	150	250	250
	RGF [24]	50.2%	95.5%	62%	29.2%	1753	645	1208	1009	765	204	408	510
	P-RGF [25]	54.2%	96.1%	73.4%	28.8%	1009	679	1169	1034	815	182	262	540
	Meta Attack [23]	20.8%	93%	59%	27%	2084	1122	2165	1863	781	651	1043	1562
	Bandits [9]	54.1%	97%	72.5%	44.9%	786	321	584	1609	56	34	32	484
	Simulator Attack [21]	72.9%	93.1%	80.7%	35.6%	**330**	233	250	1318	**30**	22	24	442
	Simulator Attack+	73.0%	74.3%	80.0%	29.7%	346	**230**	**202**	**1015**	34	**22**	**23**	**362**
TinyImageNet	NES [8]	69.5%	73.1%	33.3%	23.7%	1775	863	2908	945	850	200	1600	200
	RGF [24]	31.3%	91.8%	9.1%	34.7%	2446	1022	1619	1325	1377	408	765	612
	P-RGF [25]	37.3%	91.8%	25.9%	34.4%	1946	1065	2231	1287	891	436	985	602
	Meta Attack [23]	4.5%	75.8%	3.7%	20.1%	1877	2585	4187	3413	912	1792	2602	2945
	Bandits [9]	39.6%	95.8%	12.5%	49%	893	909	1272	1855	85	206	193	810
	Simulator Attack [21]	43%	84.2%	21.3%	42.5%	377	586	746	1631	32	148	157	632
	Simulator Attack+	41.0%	80.3%	19.0%	39.7%	**348**	**530**	**704**	**1214**	34	**146**	**154**	**582**

## Data Availability

http://www.cs.toronto.edu/~kriz/cifar.html.

## References

[1] Biggio B., Corona I., Maiorca D., Nelson B. (2013). Evasion attacks against machine learning at test time. Machine Learning and Knowledge Discovery in Databases.

[2] Goodfellow I.J., Shlens J., Szegedy C. (2014). Explaining and Harnessing Adversarial Examples. arXiv.

[3] Szegedy C., Zaremba W., Sutskever I., Bruna J., Erhan D., Goodfellow I., Fergus R. (2014). Intriguing properties of neural networks. arXiv.

[4] Madry A., Makelov A., Schmidt L., Tsipras D., Vladu A. (2017). Towards deep learning models resistant to adversarial attacks. Stat.

[5] Carlini N., Wagner D. Towards evaluating the robustness of neural networks. Proceedings of the 2017 IEEE Symposium on Security and Privacy.

[6] Chen P.Y., Zhang H., Sharma Y., Yi J., Hsieh C.J. Zoo: Zeroth order optimization based black-box attacks to deep neural networks without training substitute models. Proceedings of the 10th ACM Workshop on Artificial Intelligence and Security.

[7] Tu C.C., Ting P., Chen P.Y., Liu S., Cheng S.M. AutoZOOM: Autoencoder-Based Zeroth Order Optimization Method for Attacking Black-Box Neural Networks. Proceedings of the AAAI Conference on Artificial Intelligence.

[8] Ilyas A., Engstrom L., Athalye A., Lin J. Black-box adversarial attacks with limited queries and information. Proceedings of the International Conference on Machine Learning.

[9] Ilyas A., Engstrom L., Madry A. (2018). Prior convictions: Black-box adversarial attacks with bandits and priors. arXiv.

[10] Liu Y., Chen X., Liu C., Song D. (2016). Delving into Transferable Adversarial Examples and Black-box Attacks. arXiv.

[11] Oh S.J., Schiele B., Fritz M. (2019). Towards Reverse-Engineering Black-Box Neural Networks.

[12] Demontis A., Melis M., Pintor M., Jagielski M., Biggio B., Oprea A., Nita-Rotaru C., Roli F. Why do adversarial attacks transfer? explaining transferability of evasion and poisoning attacks. Proceedings of the 28th USENIX Security Symposium (USENIX Security 19).

[13] Huang Q., Katsman I., He H., Gu Z., Belongie S., Lim S.N. Enhancing adversarial example transferability with an intermediate level attack. Proceedings of the IEEE/CVF International Conference on Computer Vision.

[14] Chen S., He Z., Sun C., Yang J., Huang X. (2022). Universal Adversarial Attack on Attention and the Resulting Dataset DAmageNet. IEEE Trans. Pattern Anal. Mach. Intell..

[15] Orekondy T., Schiele B., Fritz M. Knockoff nets: Stealing functionality of black-box models. Proceedings of the Conference on Computer Vision and Pattern Recognition.

[16] Papernot N., McDaniel P., Goodfellow I., Jha S., Celik Z.B., Swami A. Practical black-box attacks against machine learning. Proceedings of the 2017 ACM on Asia Conference on Computer and Communications Security.

[17] Tramr F., Zhang F., Juels A., Reiter M.K., Ristenpart T. Stealing machine learning models via prediction APIs. Proceedings of the 25th USENIX security symposium (USENIX Security 16).

[18] Lee T., Edwards B., Molloy I., Su D. Defending against neural network model stealing attacks using deceptive perturbations. Proceedings of the 2019 IEEE Security and Privacy Workshops.

[19] Orekondy T., Schiele B., Fritz M. (2019). Prediction Poisoning: Towards Defenses Against DNN Model Stealing Attacks. arXiv.

[20] Xu Y., Ghamisi P. (2022). Universal Adversarial Examples in Remote Sensing: Methodology and Benchmark. IEEE Trans. Geosci. Remote Sens..

[21] Ma C., Chen L., Yong J.H. Simulating unknown target models for query-efficient black-box attacks. Proceedings of the IEEE/CVF Conference on Computer Vision and Pattern Recognition.

[22] Zhou B., Cui Q., Wei X.S., Chen Z.M. Bbn: Bilateral-branch network with cumulative learning for long-tailed visual recognition. Proceedings of the IEEE/CVF Conference on Computer Vision and Pattern Recognition.

[23] Du J., Zhang H., Zhou J.T., Yang Y., Feng J. (2020). Query-efficient Meta Attack to Deep Neural Networks. arXiv.

[24] Nesterov Y., Spokoiny V. (2017). Random gradient-free minimization of convex functions. Found. Comput. Math..

[25] Cheng S., Dong Y., Pang T., Su H., Zhu J. (2019). Improving black-box adversarial attacks with a transfer-based prior. Adv. Neural Inf. Process. Syst..

[26] Krizhevsky A., Hinton G. (2009). Learning Multiple Layers of Features from Tiny Images.

[27] Bhagoji A.N., He W., Li B., Song D. Practical black-box attacks on deep neural networks using efficient query mechanisms. Proceedings of the European Conference on Computer Vision.

[28] Cheng M., Le T., Chen P.Y., Zhang H., Yi J., Hsieh C.J. (2019). Query-Efficient Hard-label Black-box Attack: An Optimization-based Approach. arXiv.

[29] Brendel W., Rauber J., Bethge M. (2018). Decision-Based Adversarial Attacks: Reliable Attacks Against Black-Box Machine Learning Models. arXiv.

[30] Wang B., Gong N.Z. Stealing hyperparameters in machine learning. Proceedings of the 2018 IEEE Symposium on Security and Privacy (SP).

[31] Ma C., Cheng S., Chen L., Zhu J., Yong J. (2020). Switching Transferable Gradient Directions for Query-Efficient Black-Box Adversarial Attacks. arXiv.

[32] Pengcheng L., Yi J., Zhang L. Query-efficient black-box attack by active learning. Proceedings of the 2018 IEEE International Conference on Data Mining (ICDM).

[33] Papernot N., McDaniel P., Goodfellow I. (2016). Transferability in machine learning: From phenomena to black-box attacks using adversarial samples. arXiv.

[34] Guo C., Gardner J., You Y., Wilson A.G., Weinberger K. Simple black-box adversarial attacks. Proceedings of the International Conference on Machine Learning.

[35] Andriushchenko M., Croce F., Flammarion N., Hein M. (2020). Square attack: A query-efficient black-box adversarial attack via random search. Computer Vision—ECCV 2020.

[36] Chen J., Jordan M.I., Wainwright M.J. Hopskipjumpattack: A query-efficient decision-based attack. Proceedings of the 2020 IEEE Symposium on Security and Privacy (SP).

[37] Yang J., Jiang Y., Huang X., Ni B., Zhao C. (2020). Learning black-box attackers with transferable priors and query feedback. Adv. Neural Inf. Process. Syst..

[38] Inkawhich N., Liang K., Wang B., Inkawhich M., Carin L., Chen Y. (2020). Perturbing across the feature hierarchy to improve standard and strict blackbox attack transferability. Adv. Neural Inf. Process. Syst..

[39] Dong Y., Su H., Wu B., Li Z., Liu W., Zhang T., Zhu J. Efficient decision-based black-box adversarial attacks on face recognition. Proceedings of the Conference on Computer Vision and Pattern Recognition.

[40] Wu L., Zhu Z., Tai C. (2018). Understanding and enhancing the transferability of adversarial examples. arXiv.

[41] Milli S., Schmidt L., Dragan A.D., Hardt M. Model reconstruction from model explanations. Proceedings of the Conference on Fairness, Accountability, and Transparency.

[42] Ma C., Zhao C., Shi H., Chen L., Yong J., Zeng D. Metaadvdet: Towards robust detection of evolving adversarial attacks. Proceedings of the 27th ACM International Conference on Multimedia.

[43] Guo Y., Yan Z., Zhang C. (2019). Subspace attack: Exploiting promising subspaces for query-efficient black-box attacks. Adv. Neural Inf. Process. Syst..

[44] Han D., Kim J., Kim J. Deep pyramidal residual networks. Proceedings of the Conference on Computer Vision and Pattern Recognition.

[45] Yamada Y., Iwamura M., Akiba T., Kise K. (2019). Shakedrop regularization for deep residual learning. IEEE Access.

[46] Cubuk E.D., Zoph B., Mane D., Vasudevan V., Le Q.V. Autoaugment: Learning augmentation strategies from data. Proceedings of the Conference on Computer Vision and Pattern Recognition.

[47] Dong X., Yang Y. Searching for a robust neural architecture in four gpu hours. Proceedings of the Conference on Computer Vision and Pattern Recognition.

[48] Zagoruyko S., Komodakis N. (2016). Wide Residual Networks. arXiv.

[49] Jia X., Wei X., Cao X., Foroosh H. Comdefend: An efficient image compression model to defend adversarial examples. Proceedings of the of Conference on Computer Vision and Pattern Recognition.

[50] Mustafa A., Khan S., Hayat M., Goecke R., Shen J., Shao L. Adversarial defense by restricting the hidden space of deep neural networks. Proceedings of the Conference on Computer Vision and Pattern Recognition.

[51] Liu Z., Liu Q., Liu T., Xu N., Lin X., Wang Y., Wen W. Feature distillation: Dnn-oriented jpeg compression against adversarial examples. Proceedings of the 2019 IEEE/CVF Conference on Computer Vision and Pattern Recognition (CVPR).

